# Mean-variance portfolio analysis data for optimizing community-based photovoltaic investment

**DOI:** 10.1016/j.dib.2016.01.049

**Published:** 2016-02-01

**Authors:** Mahmoud Shakouri, Hyun Woo Lee

**Affiliations:** a School of Civil and Construction Engineering, Oregon State University, 220 Owen Hall, Corvallis, OR 97331, United States; b Department of Construction Management, University of Washington, 120 Architecture Hall, Campus Box 351610, Seattle, WA 98195, United States

**Keywords:** Community solar, Photovoltaic system, Portfolio theory, Energy optimization, Electricity volatility

## Abstract

The amount of electricity generated by Photovoltaic (PV) systems is affected by factors such as shading, building orientation and roof slope. To increase electricity generation and reduce volatility in generation of PV systems, a portfolio of PV systems can be made which takes advantages of the potential synergy among neighboring buildings. This paper contains data supporting the research article entitled: PACPIM: new decision-support model of optimized portfolio analysis for community-based photovoltaic investment [1]. We present a set of data relating to physical properties of 24 houses in Oregon, USA, along with simulated hourly electricity data for the installed PV systems. The developed Matlab code to construct optimized portfolios is also provided in Supplementary materials. The application of these files can be generalized to variety of communities interested in investing on PV systems.

## Specifications Table

**Table:** 

Subject area	*Civil engineering*
More specific subject area	*Energy*
Type of data	*Tables, text*
How data was acquired	*Field measurements and simulation in PVWatts*® [2]
Data format	*Raw*
Experimental factors	*Roof tilt, building orientation, azimuth, roof space*
Experimental features	*Inputs were measured in the field and used in PVWatts*® [2]*for predicting annual electricity generation*
Data source location	*Corvallis, Oregon, USA*
Data accessibility	*Data is provided in Supplementary materials directly with this article*

## Value of the data

• We propose a model to minimize hourly volatility and maximize electricity output of community-based photovoltaic systems.
• Explains the importance of diversifying PV panels among houses rather than installing a fixed number of panels on each house.
• Future research on PV valuation will be facilitated by the data included here.

## 1 Data

The physical properties of houses under study were collected in the field. Table S1 is provided as the input (azimuth, roof sloe, shading, tilt and orientation factor (TOF), roof area, and number of panels that can be installed) for simulating hourly electricity generated in PVWatts® [2]. The simulation results of PV panels for each house are given in Table S2. Table S2 is used as an input for constructing optimized portfolios. A Matlab code was developed for this purpose.

## 2 Experimental design, materials and methods

**Fig. 1:**
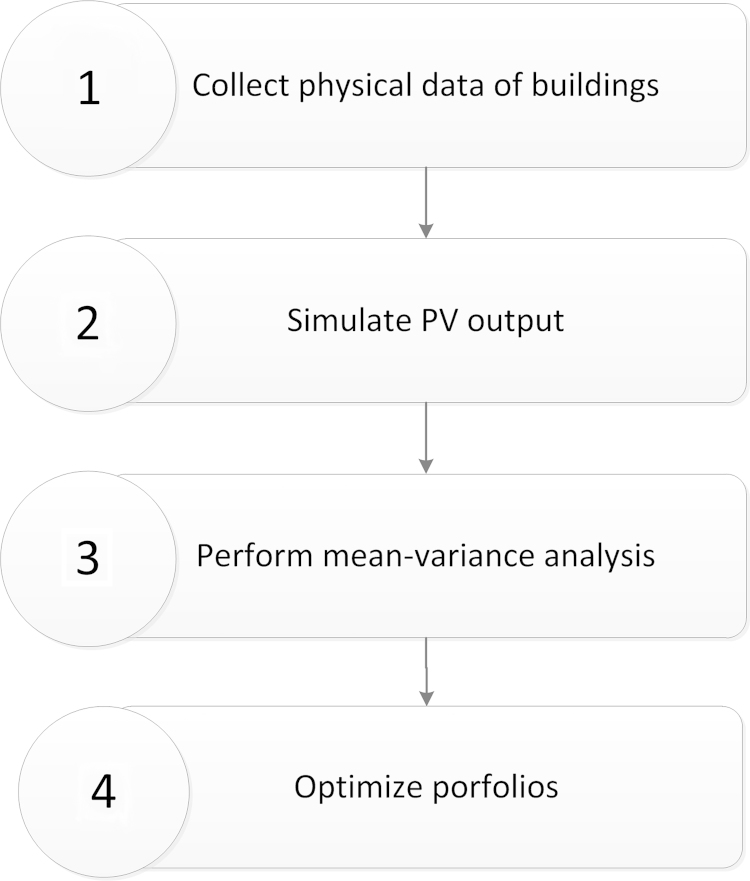
Framework of creating optimized portfolios for community-based photovoltaic investment.

Creating optimized portfolios of PV systems was carried out for a residential community consisting of 24 houses located in Corvallis, Oregon, USA [1]. The city has a has warm sunny summers, and mild wet winters with persistent overcast skies [3]. The framework that we followed to construct optimized portfolios is presented in Fig. 1. In the first step required physical data of each building was collected at site. Solmetric SunEye [4] was used to find the azimuth and amount of shading on the roof. Then, in step 2, the collected data was used as inputs for PVWatts® [2]. PVWatts estimates the annual electricity production of a PV system by using an hour-by-hour simulation over a one-year period. By default, PVWatts uses a 4 kW PV system having an array area of approximately 26 m^2^ with 15% PV panel efficiency. Since 4 kW is the most widely used system size in the U.S. residential sector, we adopted it as the baseline for the analysis. In step 3, we developed a Matlab code to create three portfolios; (1) a portfolio with maximum electricity generation; (2) a portfolio with minimum volatility in electricity generation; and, (3) a portfolio with maximum Sharpe ratio that is defined as the ratio of mean electricity generation of a portfolio to its standard deviation. Optimizing the portfolio depends on the risk-aversion level of an investor [5]. Risk-averse investors can define portfolios with low volatility but at the cost of lower electricity generation and risk-prone investors can define portfolios with high electricity generation but at the cost of high volatility. The output of step 4 is a set of weighting for each house that specifies the share of houses from total number of PV panels.

## Footnotes

Appendix A Supplementary data associated with this article can be found in the online version at doi:10.1016/j.dib.2016.01.049.
